# Persistent Metabolic Changes Are Induced by 24 h Low-Dose Lead (Pb) Exposure in Zebrafish Embryos

**DOI:** 10.3390/ijms26031050

**Published:** 2025-01-26

**Authors:** Gwendolyn Cooper, Ryan North, Tyler Hunt-Smith, James Larson, Madison Rennie, Marguerite L. Bailey, Suzanne Scarlata, Christa S. Merzdorf, Brian Bothner

**Affiliations:** 1 Department of Chemistry and Biochemistry, Montana State University, Bozeman, MT 59717, USA; gwendolyncooper@montana.edu (G.C.); jdlarson09@gmail.com (J.L.); margueritelbailey@gmail.com (M.L.B.); 2 Department of Microbiology and Cell Biology, Montana State University, Bozeman, MT 59717, USA; ryannorth@montana.edu (R.N.); tylerkhuntsmith@gmail.com (T.H.-S.); 3 Department of Chemistry and Biochemistry, Worcester Polytechnic Institute, Worcester, MA 01609, USA; mrrennie@wpi.edu (M.R.); sfscarlata@wpi.edu (S.S.)

**Keywords:** Pb exposure, development, heavy metal toxicity, metabolomics, zebrafish

## Abstract

Lead (Pb) is a heavy metal associated with a range of toxic effects. Relatively few studies attempt to understand the impact of lead on development from a mechanistic perspective. *Danio rerio* (zebrafish) embryos are a model organism for studying the developmental consequences of exposure to chemical agents. This study examined the metabolome of developing zebrafish embryos exposed to 5 ppb, 15 ppb, 150 ppb, and 1500 ppb Pb concentrations during the first 24 h post fertilization, followed by 24 h of unexposed development and harvest at 48 h. Untargeted metabolomics and multivariate analysis revealed that various Pb exposures differentially affected the embryonic metabolome. Pathway analyses showed the dysregulation of biopterin, purine, alanine, and aspartate metabolism. Inductively coupled plasma mass spectrometry demonstrated Pb accumulation in embryos. Additionally, decreases in oxidation–reduction ratios were observed in 5–150 ppb groups but not in the 1500 ppb exposure group. This finding, along with several metabolite abundances, suggests a hormetic effect of Pb concentrations on the developing zebrafish metabolome. Together, these data reveal persistent global changes in the embryonic metabolome, pin-point biomarkers for Pb exposure, unveil dose-dependent relationships, and reflect Pb-induced changes in cellular energy. This work highlights aberrant processes and persistent changes underlying low-dose heavy metal exposure during early development.

## 1. Introduction

Heavy metals like lead (Pb) are widely used in industrial settings and in diverse products including batteries, paint, and ceramics. Continued use and historic applications such as gasoline and piping have created a widespread prevalence of heavy metals in the environment. Although Pb toxicity was recognized as far back as antiquity, its use was still widespread until the 20th century due to its ductility and ease of extraction [1]. This continued and diverse use of Pb has caused the metal to be recognized as one of the most ubiquitous heavy metal contaminants present in drinking water and e-waste [2]. Ultimately, the prevalence of Pb, and thereby the high rates of acute and chronic human exposure, has resulted in a push for increased heavy metal monitoring.

The Centers for Disease Control and Prevention (CDC) in the United States has established 5 µg/dL blood Pb levels as the reference for health surveillance [3]. The United States Environmental Protection Agency (EPA) uses 15 ppb in drinking water as the action level for public supplies [3]. Though the Pb contaminant level goal is 0 ppb, many communities have Pb concentrations far exceeding the EPA limit [4,5,6]. One of the most infamous cases of Pb-contaminated water occurred in 2014 in Flint, Michigan. When the city’s drinking water source changed from Lake Huron to the Flint River, improper actions were taken to treat water, resulting in the mobilization of Pb and other heavy metals from galvanized piping. Ultimately, this resulted in many communities having Pb levels far greater than the EPA limit of 15 ppb, with samples ranging from 15 ppb to 23,100 ppb [6,7]. This is not an isolated incident—as of March 2022, half of the schools tested in Montana had high levels of Pb in drinking water, with some exceeding 7000 ppb, 1400× the EPA limit [8]. Thus, the prevalence of Pb pollution has resulted in increasing concerns for human health.

Pb exposure is associated with a range of toxic effects. According to previous studies in mice, humans, and adult zebrafish, Pb exposure causes endocrine abnormalities [9], immune toxicity [10], gut microbiota dysbiosis [11], and major neurotoxicity [12]. Pb exposure generally occurs over a lifetime, and Pb is stored in the brain, liver, kidneys, and bones [12,13]. Acute toxicity in humans is characterized by kidney, reproductive, and brain dysfunction, whereas chronic exposure causes damage to the central and peripheral nervous systems [13,14,15]. In children, larger proportions of ingested Pb are absorbed by the gastrointestinal tract compared to adults. This results in higher levels of circulating Pb, and therefore, more Pb gains access to the developing brain [13,15]. This developmental exposure results in neurotoxicity and lower IQ levels, irritability, anorexia, and lethargy [15]. In children exposed to Pb prenatally, the risk of neural tube defects appears to be elevated [16].

Pb toxicity has largely been attributed to Pb’s ability to act as a mimic for multiple divalent cations including zinc (Zn^2+^), calcium (Ca^2+^), and magnesium (Mg^2+^) [17]. This disrupts metal ion homeostasis, resulting in the generation of reactive oxygen species (ROS), the inhibition of antioxidant defenses, enzyme inhibition or stimulation, and oxidative stress [4,17]. Additionally, new research has suggested that Pb may exhibit selective binding to certain macromolecules, such as ferrochelatase and calmodulin, causing the disruption of cell proliferation [4,18,19].

These toxic effects have been documented in a zebrafish model organism. Embryonic zebrafish exposed to Pb across a range of doses show both morphological and cellular disruptions during development. High concentrations induce overt toxicities [20,21,22,23]. For example, above 10,000 ppb, survival rates decrease in a dose-dependent manner [20,21]. Zebrafish embryos exposed to doses below 1000 ppb commonly show oxidative stress responses after 48 hpf as well as spinal curvature and swim bladder abnormalities after 72 hpf [21,23,24,25]. Morphological defects are rare prior to 48 hpf for low doses of Pb [26,27]. Further, Pb exposure downregulates neurogenic gene expression and elicits changes in the expression of genes related to the BDNF signaling pathway and the GABAergic system, in addition to resulting in larval hyperactivity and memory deficits [20,22,26,27].

In mammalian models, such as mice and rats, developmental studies have mostly focused on the effects of Pb exposure on neural development, brain function, and behavior. Pb has been shown to cross the placental barrier with high efficiency [28,29,30,31,32]. High-dose (>1000 ppb) Pb exposure during the gestational periods of mammal development results in the perturbation of neuronal differentiation, neurotrophin function, neurotransmitter signaling, neuron density, and proliferation, as well as morphological impacts, including impaired spatial learning and memory, reduced body and tail length, and reduced brain and body weight [28,29,30,31,32]. These previous Pb toxicity studies have primarily focused on the physiological indicators of Pb exposure during development, while the underlying mechanisms governing small molecule profiles remain a largely unexplored area of research [33].

The toxicological effects of Pb and the internal mechanisms can be explored by using untargeted metabolomics to gain insight into changes induced in the metabolic profile. Recent metabolomic studies on the hazards of Pb exposure in adult humans show changes in oxidative stress and disruptions in energy, amino acid, and lipid metabolism [34,35]. In children exposed to Pb from an e-waste recycling plant, significant differences in metabolites from sphingolipid pathways, central carbon metabolism, and several amino acid pathways were observed [36]. Similar metabolomic observations have been noted in animal models. A recent metabolomic analysis of adult mice exposed to 10 ppm Pb-chloride showed significantly decreased vitamin E, bile acids, and glycerol 3-phosphate abundance, suggesting the dysregulation of energy-producing reactions [37]. A metabolomic analysis of Pb-exposed earthworms showed large metabolic changes in neurotransmitters, oxidative stress, and energy metabolism [38].

The majority of these studies focused on constant exposure, failing to test for metabolic changes that may persist once an organism is no longer exposed. Such persistent changes in the absence of continued exposure have the potential to cause large downstream changes during development. To better characterize the morphological, physiological, and metabolic effects of Pb exposure on embryonic development, we performed an untargeted metabolomic investigation with an emphasis on persistent changes associated with discontinued low-level Pb exposure to *Danio rerio* (zebrafish) embryos. To our knowledge, this is the first study examining the persistent effects of Pb exposure on the developing zebrafish metabolome.

## 2. Results

### 2.1. Metal and Morphological Analysis of Zebrafish Embryos After Pb Exposure

**Figure 1:**
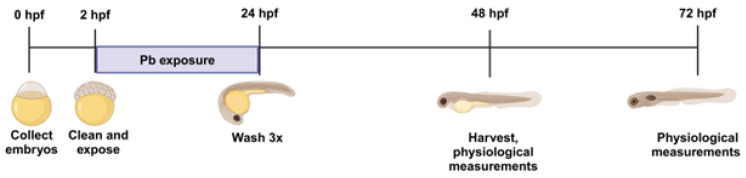
Discontinued Pb exposure model. Zebrafish embryos were collected at 0–2 hpf (hours post fertilization). Embryos were cleaned and exposed to 5 ppb, 15 ppb, 150 ppb, or 1500 ppb Pb-acetate from 2 to 24 hpf. At 24 hpf, embryos were washed and allowed to develop without Pb until 48 hpf. At this time, embryos were harvested for mass spectrometry analyses. Physiological measurements were taken at both 48 hpf and 72 hpf.

To improve our understanding of the persistent effects of Pb exposure on developing zebrafish embryos, embryos were exposed to Pb-acetate for 24 h followed by another 24 h of Pb-free development. At 48 hpf, embryos were collected for morphological analysis or were harvested for mass spectrometry-based analyses. This Pb-acetate exposure, henceforth referred to as discontinued Pb exposure, is summarized in Figure 1.

**Figure 2:**
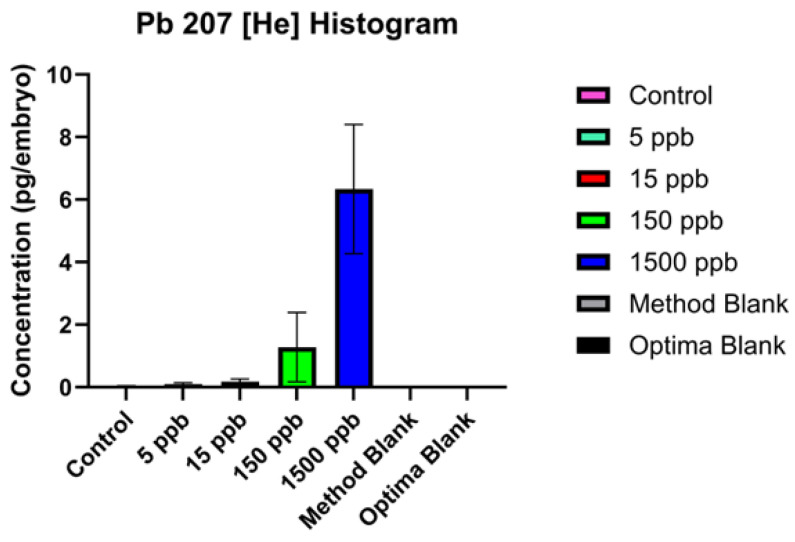
Total intracellular Pb concentrations in zebrafish embryos after discontinued Pb exposure. Concentration is normalized to pg/embryo analyzed by ICP-MS. For water concentrations (method blank and optima blank), concentrations are in ppb rather than pg/embryo as no embryos were utilized in preparation of these samples. Pb isotope 207 is reported here as instrument reports lowest detection limit (DL) and blank equivalent concentration (BEC) for this isotope. Other isotope data can be found in Supplementary Materials.

To investigate whether embryos accumulated and retained Pb during discontinued Pb exposure, elemental analysis was performed on embryos via inductively coupled plasma mass spectrometry (ICP-MS). Elemental analysis tracked Pb, Li, Be, Na, Mg, Al, K, Ca, V, Cr, Mn, Fe, Co, Ni, Cu, Zn, As, Se, Mo, Cd, Sb, Ba, Tl, Th, and U. The 5 ppb, 15 ppb, 150 ppb, and 1500 ppb exposure groups had increasing amounts of Pb accumulation (Figure 2). No other metals changed in abundance in the embryos (Supplemental Excel File, Sheet 1). Values are reported as pg/embryo. Blanks and control embryos showed no significant Pb accumulation.

A series of morphological characteristics was evaluated to determine whether Pb accumulation was affecting gross embryo development and physiology. Embryos exposed using the discontinued Pb exposure regimen were examined at 48- and 72 hpf. The heart rate measured at 48 hpf and embryo length measured at 72 hpf were not significantly different between any of the groups (*p* = 0.17 and *p* = 0.40, respectively, as determined by an Analysis of Variance (ANOVA)) (Figure S1). The visual inspections of overall development and of eye and tail development in particular did not exhibit any significant differences at 48 hpf (Figure S2) or 72 hpf.

### 2.2. Metabolic Analysis

**Figure 3:**
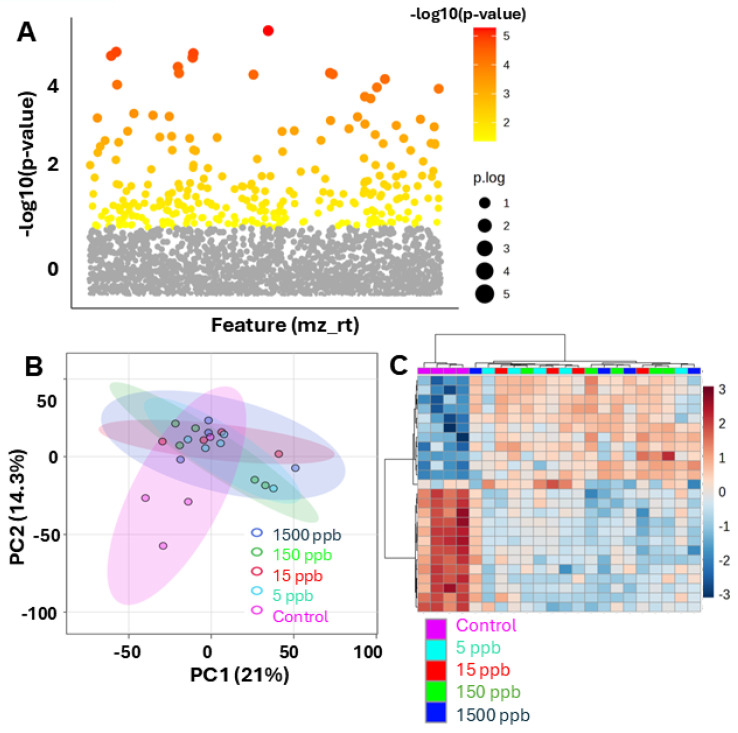
Global multivariate statistics. (**A**) One-way ANOVA of 2857 filtered metabolomic features; 275 change significantly (*p*-value < 0.05, red, yellow, and orange), and 2582 remain unchanged (gray). (**B**) Principal component analysis (PCA) score plot of metabolomic data (Purple = control; orange = 5 ppb; blue = 15 ppb; light green = 150 ppb; dark green = 1500 ppb). Ovals indicate 95% confidence intervals. Plot describes PC1 (21% of total variance) and PC2 (14.3% of total variance) coordinate pairs from each sample. (**C**) Parallel hierarchical clustering analysis (PCHA) heatmap of top 200 features, filtered by lowest *p*-value. Samples are clustered in columns, features are clustered in rows, and their intersection represents abundance of each feature in each sample relative to average. Red indicates high abundance, and blue represents low abundance.

Metabolic perturbations are far more sensitive to stress than gross physiological markers—such as heart rate and overall development [39,40]. By employing a global analysis of metabolism, subtle changes that may not be captured by traditional physiological measurements can be tracked. To investigate changes in metabolism caused by Pb exposure, untargeted metabolomic analysis was conducted on embryos subject to discontinued Pb exposure (Figure 1). In total, 2602 metabolite features were detected by LC-MS in embryo samples. To assess global differences between Pb exposure groups and controls, multivariate statistics was employed. A one-way ANOVA (false discovery rate (FDR)-adjusted *p*-value < 0.05) revealed that 275 features had significant changes in abundance, representing approximately 10% of the dataset (Figure 3A). Significant abundance changes ranged from two- to six-fold. To further visualize and interpret intergroup and intragroup variation, principal component analysis (PCA) was performed. PCA displayed a noteworthy overlap of the groups, yet the impact of Pb was visible in PC1 (Figure 3B). The groups did not appear to have dramatic global changes, as there was a significant overlap of the groups in the PCA plot. Parallel hierarchical clustering analysis (PCHA) confirmed substantial group overlap; however, it also revealed the presence of a clear metabolomic shift with Pb-treated embryos clustering away from control embryos when using the top 35 features (Figure 3C). These data demonstrate that even short term, low-dose exposure to Pb caused lasting metabolic changes. These changes included both increases and decreases in metabolite abundance in the Pb treatment groups.

### 2.3. The Metabolomic Shift Compared to Pb Concentration

**Figure 4:**
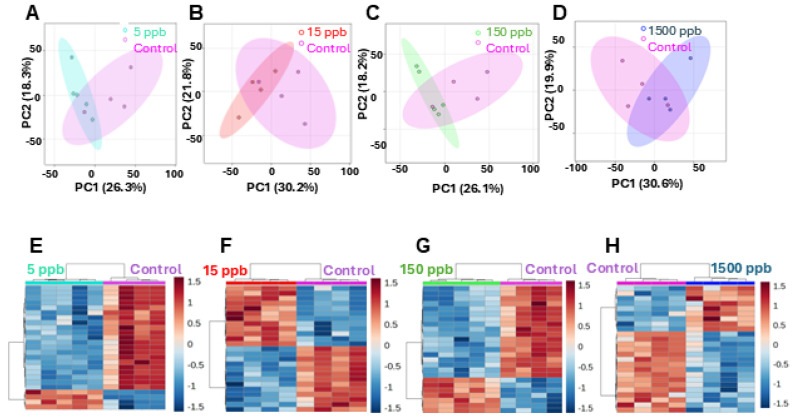
Pairwise global multivariate statistics for metabolites. (**A**) The 5 ppb group is displayed in light blue and control in pink. PC1 describes 26.3% of the total variation. PC2 represents 18.3% of the total variation. Ovals represent 95% confidence intervals. (**B**) The 15 ppb group is displayed in red and control in pink. PC1 describes 30.2% of the total variation. PC2 represents 18.2% of the total variation. (**C**) The 150 ppb group is displayed in green and control in pink. PC1 describes 26.1% of the total variation. PC2 represents 21.8% of the total variation. (**D**) The 1500 ppb group is displayed in dark blue and control in pink. PC1 describes 30.6% of the total variation. PC2 represents 19.9% of the total variation. (**E**–**H**) A PCHA heatmap of the top 35 features from each pairwise comparison. Samples are in columns, features in rows.

To determine how the various Pb concentrations differentially affected the embryonic metabolome, PCA, volcano plot analysis, and PCHA were conducted. Pairwise PCA was performed for all concentrations versus the control group. In each case, there was minimal overlap (Figure 4A–D). Volcano plot analysis identified 131 metabolite features that were significant and had higher concentrations in the 5 ppb group compared to controls and 122 metabolite features that were significant and had lower abundance in the 5 ppb group (Figure S3). PCHA revealed the perfect clustering of the control and 5 ppb groups based on the pattern of metabolite abundance, where each row represents the abundance pattern of a particular metabolomic feature (Figure 4E). Even at the lowest concentration, 24 h exposure to Pb significantly perturbed the zebrafish metabolome.

A PCHA of the pairwise comparisons revealed an interesting pattern. In the 5 ppb versus control group and 15 ppb group, there was a series of very distinct and opposite regulation trends in the top 35 metabolite features (Figure 4A,B). This can be observed in the distinct red versus blue blocks, indicating that certain features were in higher abundance in one group compared to the other. Perfect clustering was also observed in the control versus 150 ppb and control versus 1500 ppb groups. It was noted that there are less distinct blocks of similarly dysregulated features at higher Pb concentrations (Figure 4G,H). The overall lower effects of 1500 ppb Pb compared to the other exposure levels are evident in PCA clustering, as well as an overall decrease in the number of differentiating features in the volcano plot (Figure 4H and Figure S3).

**Figure 5:**
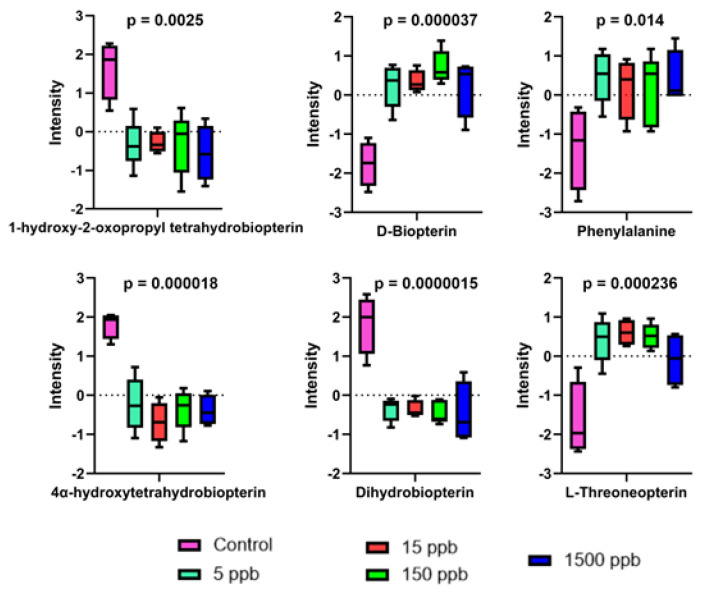
One-way parametric ANOVA of metabolites representative of biopterin metabolism. Statistically significant (FC > 2, *p* < 0.05) box and whisker plots showing normalized abundance changes in metabolites representative of biopterin metabolism across control (pink) and 5 ppb (teal), 15 ppb (red), 150 ppb (green), and 1500 ppb (dark blue) Pb-exposed groups.

**Figure 6:**
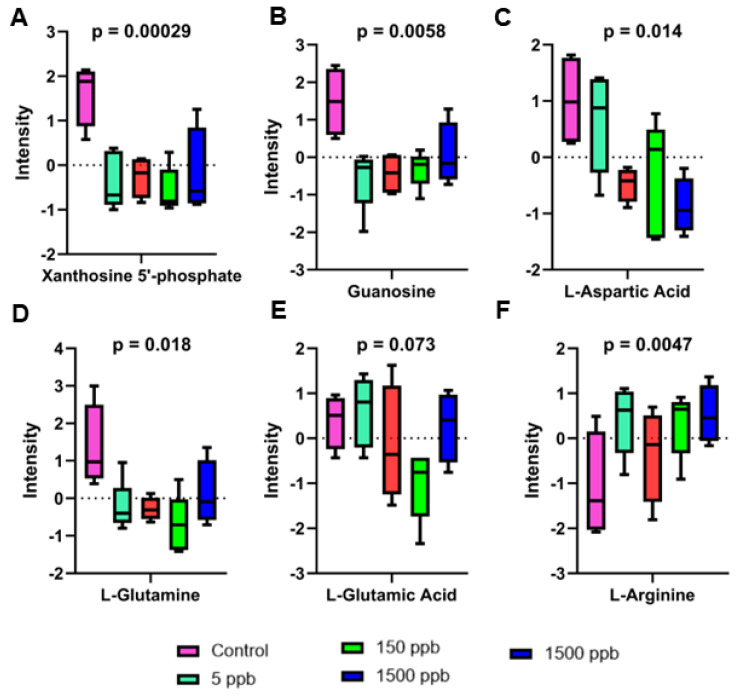
One-way parametric ANOVA of metabolites representative of purine, alanine, and aspartate metabolism. Statistically significant (FC > 2, *p* < 0.05) box and whisker plots showing normalized abundance changes in metabolites representative of purine metabolism and alanine and aspartate metabolism across control (pink) and 5 ppb (teal), 15 ppb (red), 150 ppb (green), and 1500 ppb (dark blue) Pb-exposed groups. (**A**) Xanthosine 5′-phosphate, (**B**) guanosine, (**C**) L-aspartic acid, (**D**) L-glutamine, (**E**) L-glutamic acid, (**F**) L-arginine.

Upon a further investigation of the regulated features, several metabolites were found to be shared across all Pb exposure groups. These metabolites included phenylalanine, D-biopterin, and L-threoneopterin, all of which increased in abundance with Pb exposure (Figure 5 and Figure 6). 1-hydroxy-2-oxopropyl tetrahydrobiopterin, dihydrobiopterin, 4α-hydroxytetrahydrobiopterin, xanthosine 5′-phosphate, guanosine, and glutamine decreased in abundance in all Pb exposure groups (Figure 5 and Figure 6).

The patterns of regulation revealed in the PCA, volcano, and PCHA plots indicate that significant and persistent metabolic perturbations occur in response to discontinued Pb exposure. The extent of the perturbation also appears to be disparate according to the exposure level.

### 2.4. Altered Metabolic Pathways

To further investigate the patterns of dysregulation and to pin-point biochemical pathways that distinguish the 24 h Pb-exposed from control embryos, functional pathway enrichment analyses were performed. To conduct this, feature identifications were combined with functional analysis in MetaboAnalyst to identify metabolites and their respective pathways that differentiate Pb-exposed and control groups. Pathway analysis revealed numerous pathways shared by two or three of the exposure groups (Supplemental Excel File, Sheet 2), but only three significantly dysregulated pathways were common to the 5 ppb, 15 ppb, 150 ppb, and 1500 ppb groups (*p*-value < 0.05). These included biopterin metabolism, purine metabolism, and as alanine and aspartate metabolism (Table 1).

Surprisingly, a number of pathways were only present at single exposure levels. The 5 ppb group had perturbations in five pathways: N-Glycan degradation, selenoamino acid metabolism, dimethyl-branched-chain fatty acid mitochondrial beta-oxidation, arginine and proline metabolism, and chondroitin sulfate degradation (Supplemental Excel File, Sheet 2). The 15 ppb exposure group had perturbations in five unique pathways: butanoate metabolism, fatty acid metabolism, valine–leucine and isoleucine degradation, urea cycle/amino group metabolism, and arginine and proline metabolism (Supplemental Table S1). The 150 ppb group had the greatest number of perturbed pathways, exhibiting ten pathways unique to this exposure level: glutathione metabolism, drug metabolism—cytochrome P450, TCA cycle, tryptophan metabolism, electron transport chain, sphingolipid metabolism, alkaloid biosynthesis II, limonene and pinene degradation, beta-alanine metabolism, and sialic acid metabolism (Supplemental Excel File, Sheet 2). Interestingly, the 1500 ppb exposure level has the fewest unique, perturbed pathways (xenobiotic metabolism and putative anti-inflammatory metabolite formation from EPA), indicating that the majority of metabolic changes are sensitive to lower levels of Pb.

### 2.5. Intrinsic Fluorescent Lifetime Imaging Measurements (FLIMs)

The redox homeostasis of a cell is defined as the balance between oxidants and antioxidants. Redox regulation within the cell is necessary for a plethora of reactions including cellular signaling, development, and disease [41]. This requirement means redox regulation must be highly responsive and dynamic. Cellular redox biology is difficult to study, but trends towards more oxidized states upon low-dose Pb exposure have been observed in human populations [42].

**Figure 7:**
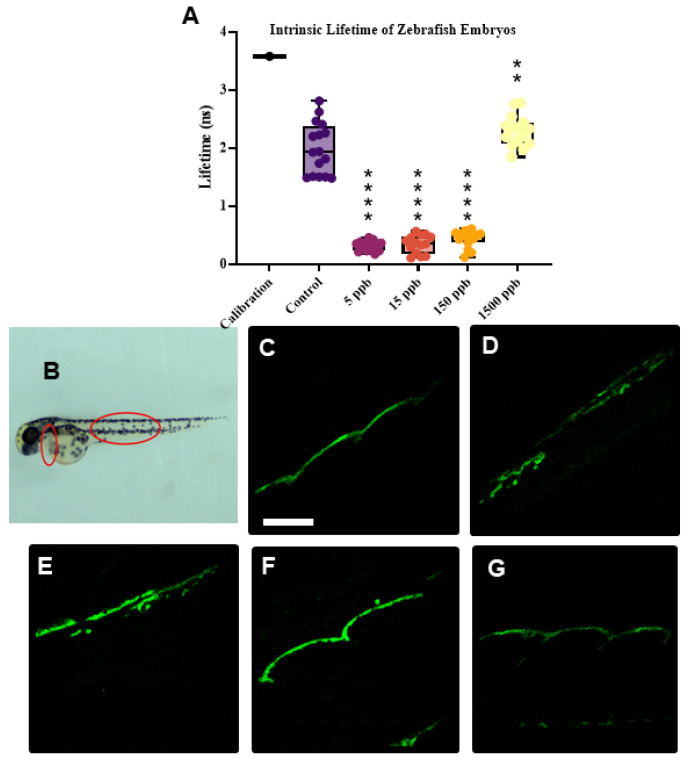
Intrinsic fluorescent lifetime imaging measurements of liver and tail regions. (**A**) Changes in lifetime of intrinsic fluorescence of Pb-treated zebrafish embryos imaged at 48 hpf, which is related to redox state. Data were visualized using GraphPad Prism and analyzed using ordinary one-way ANOVA, where (****) represents *p* < 0.0001, and (**) represents *p* < 0.01, *n* = 15–16 per condition. (**B**) Regions of adult zebrafish, circled in red, imaged to collect lifetime measurements. (**C**–**G**) Representative confocal images of zebrafish tail regions under control (**C**) and 5 ppb (**D**), 15 ppb (**E**), 150 ppb (**F**), and 1500 ppb (**G**) Pb exposure. Scale bars are 50 μm.

To investigate the impacts of Pb on the redox state, intrinsic fluorescent lifetime measurements were collected on control and 2–24 h Pb-acetate-exposed embryos (5 ppb-1500 ppb) at 48 hpf (Figure 7). In these measurements regarding the lifetimes associated with the intrinsic fluorescence values of NAD+/NADH and to a lesser extent FADH2, they report on metabolic changes associated with NADH and FAD, the two main metabolic coenzymes, which are further linked to the optical redox state [43,44]. Instead of reporting absolute values, this technique measures the ratio of oxidized to reduced fluorescent species associated with NADH and metabolic activity. With the NADH signal alone, fluorescent measurements are obtained to describe the ratio of oxidized flavoprotein signals to reduced pyridine signals, providing an intracellular report on metabolic activity with less demand on instrumentation techniques. Additionally, these coenzymes can provide information on the rate of substrate contribution to the electron transport chain (ETC), thereby reporting on cellular energy status [45]. Measurements were taken to assess the liver and tail regions (Figure 7B–D). Embryos exposed to 5 ppb, 15 ppb, and 150 ppb of Pb show a significant decrease in fluorescent lifetime compared to untreated control embryos, representing a significant drop in the redox state (Figure 7A). The drop in the fluorescent lifetime signal is consistent in both the tail and liver regions. Interestingly, at the 1500 ppb Pb exposure level, embryos did not experience a significant drop in the fluorescent lifetime signal.

## 3. Discussion

The zebrafish model system is widely used to study development and the effects of toxic substances on development. During the initial 24 hpf, zebrafish embryos complete gastrulation (10 hpf), neurulation (16 hpf), and somite formation, which establish the foundational architecture for all organ systems [46]. In humans, gastrulation occurs in the third week of gestation, directly followed by neurulation (late third and early fourth week), somite formation, and organogenesis, which initiates early heart development (early heartbeat at the end of the fourth week) and liver formation [47,48,49]. Thus, the events (and the consequences of Pb-exposure) during the first 24 h of zebrafish development are highly equivalent to those during the first month of human development. 

In order to study the effects of Pb exposure during early embryonic development, zebrafish embryos were exposed to Pb 2–24 hpf and harvested at 48 hpf to determine the persistent effects of Pb exposure on zebrafish embryos. To our knowledge, there are no other reports that examine the persistent effects of Pb exposure using a discontinued protocol (Figure 1). Most studies harvest embryos directly from Pb-containing solutions and therefore are not able to distinguish between persistent effects and the effects caused by acute exposure to Pb. These persistent effects were evaluated by untargeted metabolomic analysis, morphological assessments, and cellular redox status. We show that, although normal at a gross morphological level, the metabolomic profiles of low-dose Pb-exposed embryos are perturbed even at 5 ppb, well below the EPA limit of 15 ppb. The metabolic profiles reveal that biopterin metabolism, purine metabolism, and alanine and aspartate metabolism were dysregulated pathways at all Pb exposure levels. The data also showed the non-linear response of both the number of changed metabolites and cellular redox status in response to Pb exposure.

### 3.1. Metabolic Profiles of Pb-Exposed Embryos Are Distinct

We found that the metabolomes of control, 5 ppb, 15 ppb, 150 ppb, and 1500 ppb Pb-exposed embryos are distinct (Figure 4). Pairwise analyses examining differences between control and Pb-exposed embryos highlight that the metabolome of developing zebrafish embryos are differentially regulated depending on the level of Pb exposure. Additionally, these data reveal persistent perturbations in metabolism because embryos were only exposed to Pb during the first 24 h of development. Another observation of importance is the changes observed in the PCA, showing that biological replicates become tighter with acute stress at 5 ppb, 15 ppb, and 150 ppb which then disappears at 1500 ppb (Figure 4A–D). This is evidence of a reduction in population-level metabolic variation with acute stress. This phenotype is common in toxicology and has been observed in previous metabolomic and proteomic analyses, though the exact mechanisms that drive this phenomenon are not well understood [50,51]. It has been proposed that a metabolic bottleneck or suppression of ancillary metabolic pathways could be responsible [51]. In this study, this would mean that the presence of Pb in the embryos may activate cellular stress responses and downregulate other pathways to mitigate the physiological effects of stress.

### 3.2. Biopterin Metabolism

The homeostasis of neurotransmitters, cofactors, and gene expression are vital for the proper functioning of cells and the nervous system. For example, the lack of dopamine regulation is associated with schizophrenia, emphasizing the importance of the regulation of neurotransmitter synthetic pathways [52]. Dopamine and serotonin are critical neurotransmitters, acting on neural networks in the brain and spinal cord. Their synthetic precursors require the cofactor tetrahydrobiopterin (BH4) which also plays an important role for nitric oxide synthase (NOS) activity [53,54,55,56]. BH4 is involved in biopterin metabolism, a network of pathways that control the synthesis and regulation of this vital precursor and can also act as an intracellular antioxidant, scavenging ROS [55]. Biopterin metabolism has previously been implicated in Pb-exposed populations. In one study, pteridine metabolism was shown to be altered in workers chronically exposed to Pb, with biopterin urine concentrations up to 1.5× higher compared to unexposed participants [57]. Additionally, high levels of Pb have been associated with high levels of dopamine synthesis, implicating a potential mechanism of action for the metal’s toxicity [57].

Biopterin metabolism begins with BH4 de novo synthesis. First, GTP proceeds through multiple enzymatic transformations, resulting in the production of 7,8 dihydroneopterin, 6-pyruvoyltetrahydropterin, and BH4 [53]. Hydrolysis reactions then produce dopamine, serotonin, and tyrosine from aromatic amino acids. Additionally, phenylalanine can regulate BH4 synthesis, where high levels of phenylalanine support BH4 synthesis, and low levels slow down synthesis. It is also important to note that phenylalanine also acts in the recycling pathway of BH4, where BH4 can then act as an essential component of nitric oxide synthase. Ultimately, this results in the production of the broadly acting intracellular messenger nitric oxide (NO) [55,56]. In our metabolomic analysis, several compounds belonging to biopterin metabolism were impacted by Pb. As Pb concentration increased, D-biopterin, phenylalanine, and L-threoneopterin exhibited a relative increase in abundance in all Pb-exposed groups when compared to controls, whereas dihydrobiopterin, 4α-hydroxytetrahydrobiopterin, and 1-hydroxy-2-oxopropyl tetrahydrobiopterin all decreased in abundance in Pb-exposed groups (Figure 5). The link between Pb exposure and the upregulation of phenylalanine could reflect a change in the regulation of neurotransmitters during development. Neurotransmission and synapse development have been implicated as a result of developmental Pb exposure on the gene expression level within a zebrafish model including glutamate receptors GRIM3 and GRIA3, both of which were downregulated [58]. It has previously been shown that low-level Pb-exposure causes a reduction in dopamine in worms and microstructural changes in the dopaminergic system in healthy adult human brains [59,60]. Additionally, the upregulation of serotonin receptor expression has been observed in children with high blood Pb levels, and immunohistochemical studies have shown disruptions in the serotoninergic system [61,62]. To our knowledge, this study is the first showing that these types of changes may be reflected in the small molecule profile of developing embryos exposed to low-dose Pb concentrations.

In the final steps of the biopterin pathway, 7,8-dihydroneopterin is produced and transformed to produce neopterin, a marker of interferon ϒ, inflammation, macrophage activation, and oxidative stress [55]. Both of these compounds were identified in our metabolomic analyses, revealing a link between Pb exposure and immune system activation as a downstream result of biopterin metabolism perturbation. In short, the dysregulation of biopterin metabolism has wide-ranging impacts. The association between biopterin metabolism and the regulation of neurotransmitter levels, immune responses, liver function, and general cell metabolism and this pathway make it inherently complex to study [54,56]. It is well known that one of the key biopterin enzymes, GTP cyclohydrolase I (GTPCH), is impaired by oxidative stress [56]. One of the proposed mechanisms of Pb toxicity is the induction of oxidative stress [63,64]. When examining the relative decrease in the abundance of 1-hydroxy-2-oxopropyl tetrahydrobiopterin across Pb-exposed embryos, it is possible that the dysregulation of this metabolite contributes to alterations in the oxidative state of zebrafish embryos and, therefore, the overall dysregulation of this pathway (Figure 6A) [53]. Therefore, when taken together, the dysregulation of biopterin metabolism in Pb-exposed embryos may have large developmental consequences.

### 3.3. Purine Metabolism

Purines play a significant role in many cellular functions, including acting as building blocks for DNA and RNA and providing the energy and cofactors necessary to promote cell survival and proliferation [65]. Thus, the metabolites in this pathway represent key developmental players, and their homeostasis is critical for developmental timing [66]. Of the identified metabolites from this pathway, xanthosine 5′-phosphate, L-glutamine, L-aspartic acid, DL-glutamic acid, and guanosine were identified. Xanthosine 5′-phosphate, L-aspartic acid, L-glutamine, and guanosine exhibit a significant decrease in intensity as seen in all Pb-exposed groups when compared to the control (Figure 6). Guanosine and xanthosine 5′-phosphate are crucial for the de novo purine biosynthesis pathway, which is vital for cell proliferation and gene expression. The relative decrease in intensity of these molecules when exposed to Pb suggests perturbations in cell proliferation and survival in the developing zebrafish (Figure 6A,B). Since the embryos looked normal at the gross morphological level (Figure S1B), these changes in metabolites reflecting cell survival and proliferation were not detectable at the morphological level, thus emphasizing the need for analytical techniques like metabolomics. Some purines have more specialized roles, where adenosine and guanosine can act as neurotransmitters or as trophic agents in the nervous system [67]. Guanosine, found in high extracellular concentrations for up to a week after brain injury, is a potent in vivo neurotrophic factor known to enhance neuronal survival [68]. In this study, the relative decrease in guanosine abundance (Figure 6) may reflect decreases in neuronal survival after Pb exposure in zebrafish embryos.

Pb is also known to affect cellular energy by reducing the activity levels of mitochondrial chain enzyme complexes, leading to a decrease in ATP production and increasing mitochondrial membrane permeability [69]. Importantly, purine metabolism occurs in close proximity to mitochondria [70]. This proximity is necessary as the purine de novo pathway is energy-intensive, requiring five molecules of ATP; two molecules of glutamine and formate; and one molecule each of glycine, aspartate, and carbon dioxide to form one molecule of inosine monophosphate (IMP) [65,71]. Since Pb exposure causes an apparent decrease in energy metabolism, there is potential for a close relationship between increasing Pb exposure and perturbations in purine metabolism, resulting in an overall decrease in cellular energy.

Pb exposure has also been shown to disrupt tRNA integrity via Pb-induced cleavage and a reduction in tRNA synthetase activity [72]. This could be a key process in which alanine and aspartate metabolism is disrupted and could serve as an additional link to purine metabolism—as purines are critical for RNA repair and synthesis. Additionally, previous studies have shown the upregulation of exportin-t, responsible for exporting tRNA from the nucleus, which could be indicative of compensatory mechanisms in response to Pb perturbation [72].

### 3.4. Alanine and Aspartate Metabolism

Alanine is a non-essential amino acid produced by the reductive amination of pyruvate, and thus, it is easily produced within cells. Since transamination reactions are reversible and pyruvate is ubiquitous among cells, alanine plays an important role in pathways like glycolysis and gluconeogenesis. Aspartate serves as a neurotransmitter and maintains multiple physiological functions, such as nutritional potential, the regulation of hormones, and neuron protection [73]. The disruption of alanine and aspartate metabolism in response to oxidative stress has been noted in populations exposed to Pb via a battery recycling site, as well as in patients with non-alcoholic fatty liver disease (NAFLD), a disease in part characterized by high levels of oxidative stress [74,75]. Oxidative stress and ROS stimulate glutathione (GSH) synthesis from cysteine, glycine, and glutamate, which are produced from the transamination of alanine and aspartate.

As one of the metabolites identified with high confidence in our data, glutamic acid, a proxy for glutamate, exhibited a decrease in relative abundance as Pb concentration increased, with a slight recovery observed in the 1500 ppb group (Figure 6E). The relative decrease in glutamic acid concentrations is a consistent finding among Pb-exposed populations; however, the apparent recovery observed in the 1500 ppb group is novel [35,76]. The decrease in glutamic acid, a key requirement for the production of GSH, underscores the impact Pb exposure has on cellular oxidative management pathways. In the present study, embryos were only exposed to Pb from 2 to 24 hpf and then were left to develop in the absence of Pb to 48 hpf; thus, the changes observed exclude acute changes and reflect persistent changes induced by Pb exposure. The slight increase in glutamic acid abundance between 150 ppb and 1500 ppb exposed embryos could indicate the activation of cellular responses and the downregulation of other pathways to cope with the physiological effects of Pb [51]. Additionally, it could be representative of a hormetic response in which glutamic acid is inhibited until a certain threshold of Pb exposure is met, and then the reverse response can be observed [17].

Glutamate has been shown to exhibit hormetic relationships in rats exposed to Pb. In one study, rats were observed to exhibit U-shaped dose–effect relationship curves [77]. This type of hormetic response to Pb has been documented for calmodulin and phospholipase C activity, as well as chlorophyll concentration and NO content in plants, but could be true for other enzymes and their respective metabolite pools as well [17,78,79]. Though it is an intriguing theory that appears to be corroborated by this finding and the FLIM data, further research is required to understand this response.

Glutamate also plays a significant role as a neurotransmitter. Like all other neurotransmitters, glutamate relies on cations, namely Ca, for synaptic vesicle release. Pb can mimic Ca in several ways. It can be carried into cells via Ca channels and be transported by and substitute Ca as the ligand for Ca-ATPase, and since the ionic radius of Pb is similar to that of Ca (1.19 Å vs. 1.00 Å), it can act as a functional ion mimic of endogenous ions at intracellular binding sites [80,81,82]. By mimicking Ca, glutamate release is reduced and decreases the activation of postsynaptic receptors [17]. In turn, this decreases postsynaptic potential and signal transmission. Glutamate has been implicated in Pb neurotoxicity, and identification in our study highlights the multifaceted nature of Pb toxicity [63,64,76]. Pb accumulation and toxicity following exposure have been observed in other studies and can be affected by the presence of other metals, primarily Cd, Ti, Ni, and Zn [83,84]. Interestingly, the ICP-MS data collected did not show any significant changes in Ca or any other metal concentration within the embryos upon Pb exposure (Figure S4). This is in contrast to a recent study where exposure to certain heavy metals caused changes in the abundance of other metals, compounding toxic effects [85]. Therefore, in this model, toxicity is likely to be due to the direct effects of Pb rather than the secondary regulation of other metals.

### 3.5. Oxidative Stress

As a direct probe of oxidative stress, intrinsic fluorescent lifetime imaging measurements (FLIMs) were taken. FLIM is an emerging optical technique for sensing and quantifying metabolism which reports directly on the mitochondrial oxidation–reduction state by measuring the ratio of oxidized to reduced proteins from intrinsic NADH measurements. This is made possible by measuring the fluorescent decay parameters of several endogenous fluorophores: NAD+/NADH and, to a lesser extent, FADH2 [86]. Since multiple pathways including NF-kB and MAPK are highly sensitive to oxidative stress and are affected by Pb exposure, a direct analysis of the effects of discontinued Pb exposure on the oxidation–reduction states of zebrafish embryos was of interest [87].

In this study, we found that zebrafish embryos exposed to 5 ppb, 15 ppb, and 150 ppb Pb all exhibited decreases in fluorescent lifetimes and therefore lower oxidation–reduction ratios (Figure 7). This is indicative of a decrease in energy production metabolic activity since tissues found in the liver and tail regions exhibit less NADH autofluorescence, indicating less oxidized and reduced species in these tissues. Since this method directly probes the signal from mitochondria in the tissues found in the liver and tail regions imaged (Figure 7B), these findings support the observations of purine metabolism and energy production being dysregulated.

### 3.6. Hormetic Responses

The 1500 ppb exposed embryos did not exhibit a decrease in fluorescent lifetimes, suggesting that the oxidation–reduction ratios of these embryos were more similar to those of the control rather than the other Pb-exposed embryos (Figure 7A). These U-shaped patterns, or hormetic patterns, can be indicative of a toxicological phenomenon in which exposure to an agent causes a biphasic dose response [88]. More specifically, this response is observed by lower exposure levels causing significant inhibitory effects in an organism and higher exposure levels actually causing beneficial or stimulatory effects [88]. This U-shaped response to the oxidation state in response to Pb concentration was also observed in response to the duration of Pb exposure [24]. At a dose of 100 ppb of Pb, 48 h of exposure led to an increase in the relative ratio of ROS per larva, whereas 72 h of exposure resulted in a decrease, suggesting a nuanced response to both the concentration and duration of Pb [24]. Hormetic responses can also be observed in the violin plots of several key metabolites identified within this study, including glutamine and glutamic acid. Hormetic effects have been previously reported in Pb-exposed groups; however, to our knowledge, these effects have never been studied at the metabolomic level within a model for development [17]. Interestingly, heavy metal-induced hormetic effects have been observed with diverse models [79,89,90]. While these observations are common, the mechanisms driving this non-linear response are unclear. Some metallothioneins are known to detoxify metals, and their expression can be induced by cadmium in human T-cells [91]; however, the mechanism driving the Pb-induced hormetic response observed in zebrafish embryos herein remains unknown. Further research would benefit from an investigation of proteins known to detoxify heavy metals and the mechanisms that govern the detoxification process. When taken together, the persistent changes induced by discontinued Pb exposure on developing embryos may first present as subtle, but in reality, they are quite complex.

## 4. Materials and Methods

### 4.1. Zebrafish Maintenance and Exposure

Adult *Danio rerio* (zebrafish) were maintained under standard conditions in fish water at a temperature of 28 (+/−1 °C). Embryos were collected and cleaned with 0.3× Danieau solution at 0–2 h post fertilization (hpf) for Pb exposure as described below. Embryos were incubated at 28 °C with a 14 h light–10 h dark cycle. All procedures were approved by the Institutional Animal Care and Use Committees at Montana State University—Bozeman.

At 2 hpf, each clutch was divided into one control group and four lead exposure groups (5, 15, 150, and 1500 ppb) of 60 embryos each. Lead(II) acetate trihydrate (ThermoFisher Scientific, Loughborough, UK) was added to each exposure dish to the desired levels in 0.3× Danieau solution. All embryo dishes were placed in an incubator at 28 °C overnight.

At 24 hpf, the embryos from each dish were washed with Danieau 3 times and placed into a new dish with fresh 0.3× Danieau without Pb. The embryos were then returned to the incubator for another 24 h. At 48 hpf, the embryos were collected for morphological analyses or were harvested for mass spectrometry-based analyses (metabolomics and total metal analysis). The Pb-acetate exposure regimen (Figure 1) will henceforth be named “discontinued Pb exposure”.

For mass spectrometry analyses, 30 embryos from each experimental or control group were placed into an Eppendorf tube, and any remaining solution was removed using a gel-loading pipette tip. Embryos were then frozen at −80 °C until the subsequent analysis. Analysis was performed on 4–5 biological replicates, all consisting of 30 embryos per tube.

### 4.2. Embryonic Imaging

The morphological development of embryos was followed from 48 to 72 hpf as described by Kimmel et al. (1995) [46] using a Zeiss Discovery V8 microscope (Zeiss AG, Oberkochen, Germany) equipped with a JenOptics Arktur (Jenoptik, Jena, Germany) camera. Heart rate was measured using the live feed. Embryo length was calculated utilizing ImageJ version 1.54d https://imagej.nih.gov/ij/ (accessed 20 April 2023) per established protocols [92].

### 4.3. Pb Concentrations Determined by Inductively Coupled Plasma Mass Spectrometry (ICP-MS)

To quantify metal accumulation in the embryos, samples consisting of 30 whole embryos per biological replicate were first digested with two initial rounds of Optima-grade nitric acid followed by two rounds of hydrogen peroxide digestion. All digestions occurred at 95 °C and went to dryness, as described by Thomason et al. [93]. Method blanks, defined as fresh tubes containing solely nitric acid and hydrogen peroxide, were processed identically to the tubes containing embryos to test for background metal accumulation during sample collection. Optima blanks were generated with the solvents used during sample processing to test for background metal accumulation. Samples were then resuspended in 4 mL of 2% Optima-grade nitric acid. Once resuspended, samples were filtered using an Acrodisc 32 mm syringe filter with a 0.8/0.2 µm Supor membrane (Pall Corporation, Port Washington, NY, USA). Filtrates were analyzed on an Agilent 7800 ICP-MS equipped with a MicroMist nebulizer (Agilent, Santa Clara, CA, USA) and an SPS4 autosampler (Agilent, Santa Clara, CA, USA).

Metal concentrations were determined using an eight-point standard curve generated from a serial dilution of a commercially available environmental calibration standard (CPI International, Santa Rosa, CA, USA). An internal standard mix (Agilent, Santa Clara, CA, USA) was added to the samples using a T-junction immediately before the nebulizer to account for instrumental drift and matrix effects. ICP-MS parameters were auto-tuned using an ICP-MS tuning solution (Agilent, Santa Clara, CA, USA). Data acquisition and analysis were conducted using Agilent MassHunter 4.6 (version C.01.06).

### 4.4. Sample Preparation for Metabolomic Analysis

Thirty whole embryos per biological replicate were harvested for metabolite extraction. A total of 200 µL of cold acetone was added to the samples prior to homogenization to assist in protein precipitation and metabolite extraction. Homogenization was achieved using a water bath sonication system (Cole-Parmer, Vernon Hills, IL, USA) for 15 min at 15 °C. Samples were then placed in a −80 °C freezer overnight. The following morning, samples were centrifuged at 16,100× *g* for 10 min at 4 °C. The supernatant was harvested and dried via a vacuum concentrator to evaporate solvent and isolate metabolites. Prior to analysis using a mass spectrometer, dried metabolites were resuspended in 100 μL of 1:1 acetonitrile–water. At this time, pooled samples, consisting of 5 µL of resuspended metabolites from each sample, were generated. Two quality control samples were created to account for any potential contamination: one that underwent metabolite extraction using only solvents and one that contained only mass spectrometry-grade water.

### 4.5. LC-MS/MS Metabolite Analysis

Following metabolite extraction, all samples were analyzed by liquid chromatography–mass spectrometry (LC-MS) using an Acquity UPLC plus coupled through an electrospray ionization source to a Waters Synapt XS (Waters, Milford, MA, USA). Metabolite separation was achieved using a Cogen Diamond Hydride HILIC column (150 × 2.1 mm) at a flow rate of 400 µL/min. The solvents used were 95% water 5% acetonitrile with 0.1% formic acid (solvent A) and 95% acetonitrile 5% water with 0.1% formic acid (solvent B). The 19 min elution gradient consisted of 95% to 25% solvent B over 12 min, a 5 min washconsisted of 25% solvent B, and each run began with 2 min of washing. Quality control blanks were injected every 12 samples throughout the run to track spectral drift and to assess LC-MS performance. All samples underwent standard MS1, and pooled samples underwent liquid chromatography tandem mass spectrometry (LC-MS/MS) with a constant energy ramp of 20–50 V. Data from all samples were inspected manually to determine whether any issues arose during the run.

### 4.6. Global Metabolomic Profiling

LC-MS data consisting of mass-to-charge ratios (*m/z* values), relative ion abundances, and retention times were processed using Progenesis QI (Nonlinear Dynamics, Newcastle, UK) and MetaboAnalyst. To correct for non-normal distributions, all data underwent quantile normalization, were log-transformed, and auto-scaled (mean-centered and divided by the standard deviation of each variable).

For the statistical analyses and data visualization of metabolomic profiles, MetaboAnalyst was utilized. Specifically, principal component analysis (PCA) and partial least square discriminant analysis (PLS-DA) were used to visualize the presence, absence, or overlap between Pb-exposed and control metabolomic phenotypes. Fold change was used to measure relative changes in the concentration of metabolite features between groups. Volcano plots were used to assess the magnitude and significance of change. When used together, these analyses pin-point the populations of metabolites that are differentially regulated between groups. Hierarchical clustering analyses (HCA) were performed to distinguish the clusters of coregulated metabolite features. Pathway enrichment analyses using functional analysis in MetaboAnalyst were utilized to predict the networks of functional activity and derive the biological relevance of the inputted metabolite features.

For metabolite identification from LC-MS/MS data, Progenesis QI (Nonlinear Dynamics, Newcastle, UK) was utilized. All MS1 and MS2 centroided data were imported, aligned, and peak-picked. The Human Metabolome Database (HMDB) and an in-house metabolite library (Mass Spectrometry Library of Standards, IROA Technologies, Ann Arbor, MI, USA) were utilized to compare acquired and theoretical fragmentation for identifications. Metabolite identification required a Progenesis score of greater than 30 using mass error, isotope distribution, and fragmentation. For mass accuracy, parts per million (ppm) errors over 20 ppm were excluded.

### 4.7. Fluorescence Lifetime Imaging Measurements (FLIMs)

Live embryos at 48 hpf were chosen randomly for each condition and embedded in 1% low-melt agarose in 0.3× Danieau solution. To embed them, embryos were placed in 35 mm Petri dishes affixed with a poly-D-lysine treated glass-bottom (Mat-Tek, Ashland, MA, USA). Once placed, excess 0.3× Danieau was removed, and 100 µL of cooled 1% agarose was placed on top of embryos. Once agarose was set, dishes were covered with 1 mL of 0.3× Danieau to remain hydrated while imaging.

Phase modulation FLIMs were performed on dual-channel confocal fast FLIM (Alba version 5, ISS Inc., Wilmington, NC, USA) using a two-photon titanium–sapphire laser and a Nikon Eclipse Ti-U inverted microscope. The lifetime of the laser was calibrated each time before conducting experiments by measuring the lifetime of Atto 425 in water with a lifetime of 3.61 ns at 80 MHz. Embryos were excited at 740 nm for intrinsic measurements, and emission spectra were collected through a 525/50 bandpass filter and 60× objective. For each measurement, the data were acquired using FastFLIM mode on VistaVision software 64-bit version 4.2.542.

## 5. Conclusions

The persistent effects of discontinued Pb exposure on developing zebrafish embryos highlight the multifaceted mechanisms by which Pb exerts its toxicity. Morphologically, zebrafish embryos appeared to develop normally; however, untargeted metabolomics revealed significant metabolic changes, which included perturbations in biopterin metabolism, which may reflect changes in neurotransmitter regulation and immune response and an increase in oxidative stress. Changes in purine metabolism were also observed, suggesting changes in cellular energy and cell proliferation. Alanine and aspartate metabolism also exhibited changes in response to Pb exposure. These changes are suggestive of increased oxidative stress as well as decreases in energy-producing pathways. Additionally, low-dose Pb exposure induced a hormetic response in several small molecules as well as intrinsic fluorescent lifetimes. Taken together, these findings help us understand the biochemistry behind Pb exposure at the embryonic level and support the development of dose-dependent Pb relationships.

## Figures and Tables

**Table 1:** Metabolic pathways associated with lead exposure. Significantly (P(Gamma) < 0.05) dysregulated metabolic pathways associated with every Pb-exposed group.

Pb Concentration		5 ppb	15 ppb	150 ppb	1500 ppb
**Biopterin metabolism**	Pathway total	26	26	26	26
	Hits total	9	9	11	9
	Significant hits	7	6	11	6
	*p* (gamma)	0.01	0.0073	0.0075	0.0051
**Purine metabolism**	Pathway total	80	80	80	80
	Hits total	32	32	27	32
	Significant hits	16	15	11	12
	*p* (gamma)	0.012	0.0066	0.01	0.0056
**Alanine and aspartate metabolism**	Pathway total	20	20	20	20
	Hits total	10	10	9	10
	Significant hits	5	7	4	4
	*p* (gamma)	0.036	0.0066	0.025	0.015

## Data Availability

The raw data supporting the conclusions of this article will be made available by the authors on request.

## Supplementary Materials

The following supporting information can be downloaded at https://www.mdpi.com/article/10.3390/ijms26031050/s1.
