# Supplementary material for: Persistent Metabolic Changes Are Induced by 24 h Low-Dose Lead (Pb) Exposure in Zebrafish Embryos
Source: Int J Mol Sci. 2025 Jan 26;26(3):1050. doi: 10.3390/ijms26031050 (PMC11817773; doi:10.3390/ijms26031050)
Supplement: Supplementary file 1 [file ijms-26-01050-s001.zip › Supplemental Materials.pdf]

**Supplementary Material**

**Title:** Persistent Metabolic Changes Are Induced by 24 h Low-Dose Lead (Pb) Exposure in Zebrafish Embryos

**Authors:** Gwendolyn Cooper [1], Ryan North [2], Tyler Hunt-Smith [2], James Larson [1], Madison Rennie [3], Marguerite L. Bailey [1], Suzanne Scarlata [3], Christa S. Merzdorf [2,*] and Brian Bothner [1,*]

[1] Department of Chemistry and Biochemistry, Montana State University, Bozeman, MT 59717, USA

[2] Department of Microbiology and Cell Biology, Montana State University, Bozeman, MT 59717, USA

[3] Department of Chemistry and Biochemistry, Worcester Polytechnic Institute, Worcester, MA 01609, USA

* Correspondence: merzdorf@montana.edu and bbothner@montana.edu

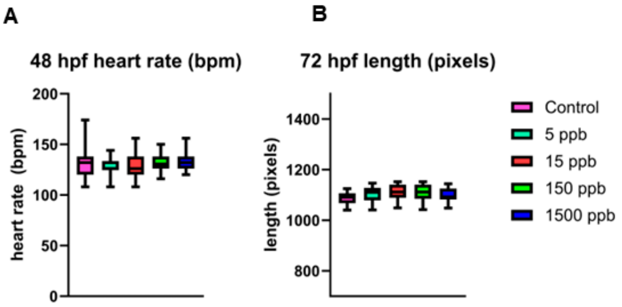


**Figure S1: Morphological data of lead exposed and control embryos.** (**A)** Box and whisker plots displaying heart rate (bpm) of control, 5ppb, 15ppb, 150ppb, and 1500ppb lead exposure groups. Two-tailed t-test assuming equal variances show no significant differences between the control and experimental groups. (Control/5ppb p = 0.21457; Control/15ppb p = 0.22205; Control/150ppb p = 0.717882; Control/1500ppb p = 0.685469). As determined by an ANOVA, no significant differences can be observed in these data (p = 0.169963). (**B)** Box and whisker plots showing embryo length (pixels) of the control and lead exposed groups at 72 hpf. Two-tailed t-tests assuming equal variance show no significant differences between control and experimental groups. (Control/5 ppb p = 0.244808; Control/15 ppb p = 0.12093; Control.150 ppb p = 0.31696; Control/1500 ppb p = 0.398624951). As determined by an ANOVA, no significant differences can be observed in these data (p = 0.401202).

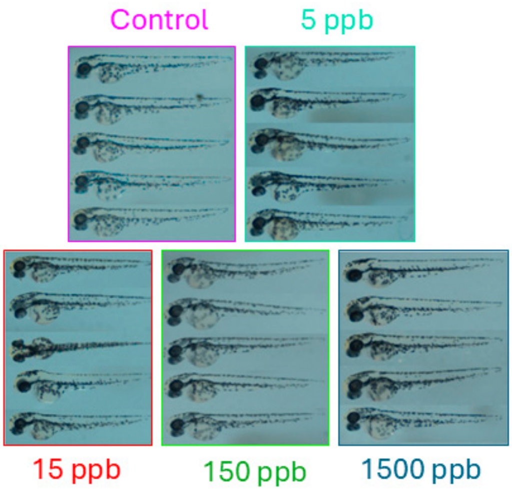


**Figure S2: Morphological images of control and lead exposed embryos.** Visual inspection of control and Pb exposed 48 hpf embryos showed no significant differences in head, eye, and tail development as a result of 5 ppb, 15 ppb, 150 ppb, or 1500 ppb Pb exposure.

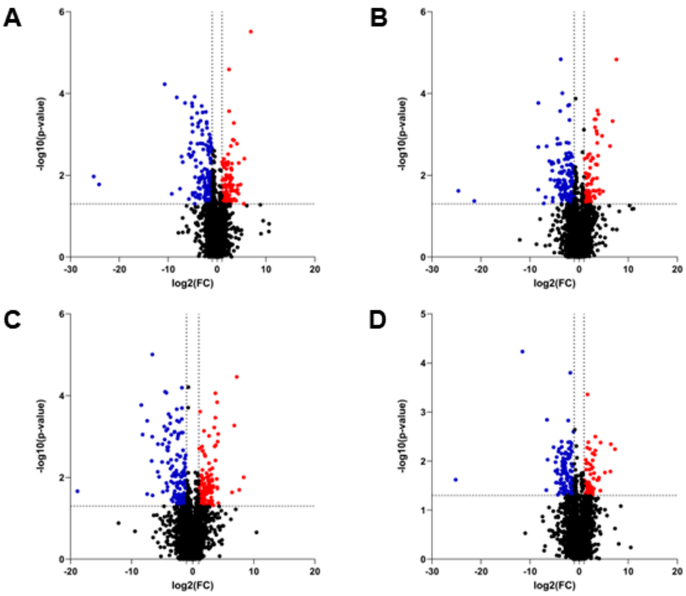


**Figure S3: Volcano plots of pairwise comparison.** Log transformed fold change values (Log2(FC)) on the x-axis are compared to statistical significance (-log10(p)) on the y-axis. Directionality of the comparison is 5 ppb/control, 15 ppb/control, 150 ppb/control, and

1500 ppb/control. (**A)** 5 ppb/control volcano plot. Blue dots represent a lower abundance in 5 ppb (171) and red indicate a higher abundance in 5 ppb (124). Black dots represent features below the threshold of statistical significance and fold change (2558) (p-value < 0.05, FC > |2|). (**B)** 15 ppb/control volcano plot. Blue dots represent a lower abundance in 15 ppb (138) and red indicate a higher abundance in 15 ppb (82). Black dots represent features below the threshold of statistical significance and fold change (2634) (p-value < 0.05, FC > |2|). (**C)** 150 ppb/control volcano plot. Blue dots represent a lower abundance in 150 ppb (141) and red indicate a higher abundance in 150 ppb (128). Black dots represent features below the threshold of statistical significance and fold change (2586) (p-value < 0.05, FC > |2|). (**D)** 1500 ppb/control volcano plot. Blue dots represent a lower abundance in 1500 ppb (114) and red indicate a higher abundance in 1500 ppb (70). Black dots represent features below the threshold of statistical significance and fold change (2670) (p-value < 0.05, FC > |2|).

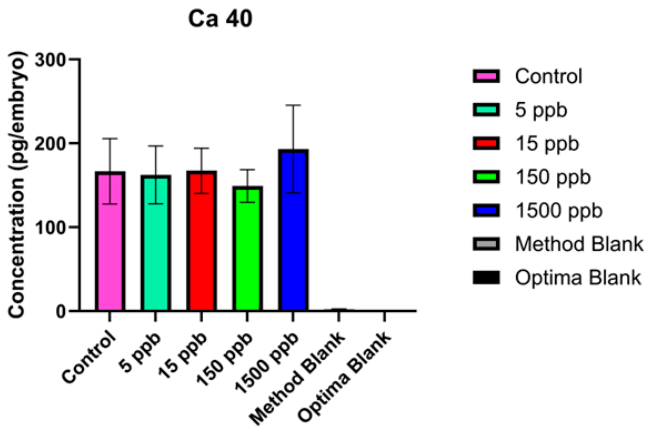


**Figure S4: Total intracellular Ca concentrations normalized to pg/embryo analyzed by ICP-MS.** For water concentrations (method blank and optima blank) the concentrations are in ppb rather than pg/embryo as no embryos are utilized in preparation of these samples. Calcium isotope 40 was used based on the lowest BEC and DL obtained from the standard curve. Other isotope data can be found in Supplemental.
